# Anti-N-methyl-D-aspartate Encephalitis Concomitantly with Tall-cell Variant Papillary Thyroid Carcinoma

**DOI:** 10.7759/cureus.5415

**Published:** 2019-08-18

**Authors:** Ahmad Mahadeen, Naresh Mullaguri, Pravin George, Laura Rabinowitz, Christopher R Newey

**Affiliations:** 1 Neurology, Cleveland Clinic, Cleveland, USA; 2 Pathology, Cleveland Clinic, Cleveland, USA

**Keywords:** nmda encephalitis, papillary thyroid cancer, autoimmune encephalitis, fluorodeoxy glucose positron emission tomography (fdg-pet), seizures

## Abstract

Anti-N-methyl-D-aspartate (NMDA) encephalitis is an autoimmune-mediated process characterized by psychosis, seizures, dyskinetic movements, and autonomic instability. At least half of the reported cases are paraneoplastic, particularly associated with an ovarian teratoma. None have been reported to be associated with thyroid tumor. We present a case of anti-NMDA encephalitis concomitantly occurring in setting of papillary thyroid carcinoma in a woman who presented with headaches, myalgia and somnolence mimicking meningoencephalitis.

A 29-year-old African female presented with fever, headache, myalgia, somnolence and behavioral changes. Initial evaluation was significant for lymphocytic pleocytosis with normal glucose and protein. She was started on broad spectrum empiric antibiotics. Despite antibiotics, she continued to have worsening encephalopathy, hallucinations, epileptic seizures, and multifocal dyskinesias involving the face and extremities with no electroencephalogram correlate. Extensive infectious workup was unremarkable. Whole-body CT with contrast and ovarian ultrasound were unremarkable for malignancy. Serum auto-antibodies to the NMDA receptor and thyroid peroxidase were detected. She was treated with high-dose intravenous steroids, plasmapheresis, intravenous immunoglobulin, and rituximab with no clinical or serological response. Fluorodeoxyglucose positron emission tomography (FDG-PET) showed a hypermetabolic thyroid nodule. Fine needle aspiration of the nodule revealed papillary thyroid carcinoma. She underwent total thyroidectomy and pathology showed two foci of tall-cell variant papillary thyroid carcinoma. Serological and clinical response followed shortly after tumor resection. The NMDA receptor stain of the papillary thyroid carcinoma was nonreactive.

We describe the coincidentally co-occurrence of NMDA encephalitis in a patient with papillary thyroid carcinoma. This case highlights the importance of presumed cases of non-paraneoplastic NMDA encephalitis, FDG-PET may help in detecting occult malignancies.

## Introduction

Anti-N-methyl-D-aspartate receptor (NMDA) encephalitis is a rare autoimmune-mediated condition that affects predominantly young women [1]. In majority of cases, patients have associated malignancy, particularly an ovarian teratoma [1-3]. Clinical presentation is characterized by mood disorders, psychosis, hyperkinetic movements, seizures, autonomic instability, and encephalopathy [1,4]. Management includes immunosuppression and screening with removal of malignancy, if present [1, 5]. Fluorodeoxyglucose positron emission tomography (FDG-PET) can increase the yield of detecting occult malignancy if other imaging studies are unremarkable [6-7]. Immunosuppression is required in all cases [1]. Additional cancers have been reported to cause NMDA encephalitis including neuroendocrine tumors, small cell lung cancer, and thymoma [8]. None have reported NMDA encephalitis associated with thyroid tumor [9]. We present a case of NMDA encephalitis concomitantly occurring in a patient with newly found tall-cell variant papillary thyroid carcinoma.

## Case presentation

A 29-year-old previously healthy Nigerian female presented to her primary care physician for a week duration of a sudden onset, frontal headache and diffuse myalgias. She was sent home with treatment for her headache. However, over the next three days, she was noted to be febrile, encephalopathic with unexplained behaviors and unintelligible speech. She subsequently presented to her local emergency department for the worsening illness and was admitted.

On initial examination, she was febrile, profoundly encephalopathic and displayed dyskinesias of the face, mouth, tongue and upper extremities. Cerebrospinal fluid (CSF) analysis on admission had 40 red blood cells (RBC) per microliter (uL) (normal 0-5/uL), lymphocytic pleocytosis with 259 white blood cells (WBC) per uL (normal 0-5 uL), normal protein (normal range 15-45 mg/dL) and normal glucose (normal range 40-70 mg/dL). She was initially started on empiric broad-spectrum antibiotics (acyclovir, ceftriaxone and vancomycin) and dexamethasone while awaiting CSF results. Computed tomography (CT) of the head was unremarkable. Magnetic resonance imaging (MRI) of the brain with intravenous contrast showed a small focus of susceptibility artifact in the right parietal lobe which was interpreted as a sequela of remote hemorrhage. She continued to have progressive confusion, agitation, and hallucinations. She developed generalized tonic-clonic seizures requiring treatment with valproic acid, levetiracetam, lacosamide, oxcarbamazepine, phenobarbital, and eventually continuous midazolam infusion. She developed hyperthermia despite broad-spectrum antibiotics. Repeat lumbar puncture on day 6 of admission (10 days from symptom onset) demonstrated lymphocytic pleocytosis (217 WBC/uL), elevated RBCs (40/uL) with normal protein and glucose. Oligoclonal bands were present in the CSF along with an elevated Immunoglobulin G (IgG) index at 0.82 (normal 0-0.61) and synthesis rate was 4.4 mg/day (normal 0-3 mg/d). Cytology was negative. Infectious workups for bacterial, fungal and viral encephalitis were unremarkable. Acyclovir was discontinued after six days of treatment. She was eventually transferred to our tertiary care hospital for a second opinion regarding undetermined meningoencephalitis and refractory status epilepticus after 23 days from symptom onset.

**Figure 1:**
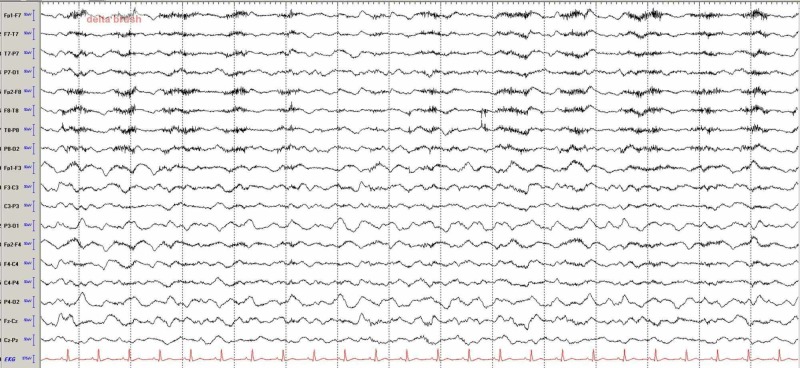
Electroencephalography (EEG) showing high amplitude continuous rhythmic delta slowing. Overriding faster frequencies can be seen on the delta slowing consistent with delta brush.

On admission, she had continued dyskinetic movements with rhythmic facial and bilateral upper and lower extremity dyskinesias/tremors/rhythmic flexion. Electroencephalography (EEG) monitoring for 72 hours showed ‘high amplitude delta activity with overriding fast (extreme delta brush pattern) but no electrographic seizures (Figure 1).

Her antiepileptic medications were subsequently weaned.

Repeat MRI of the brain with and without intravenous contrast was unremarkable. CT of the chest, abdomen and pelvis and pelvic ultrasound showed no signs of malignancy or infection. Paraneoplastic and autoimmune workup including serum NMDA titers were drawn. Repeat cerebrospinal fluid analysis was remarkable for elevated RBC (153/uL), lymphocytic pleocytosis (53 WBC/uL), CSF/serum glucose ratio of 0.46 with normal protein. CSF neopterin was slightly elevated at >300 nmol/L (normal range 8-28 nmol/L). IgG index was 2.91. CSF viral, bacterial, and fungal workup was negative.

**Figure 2:**
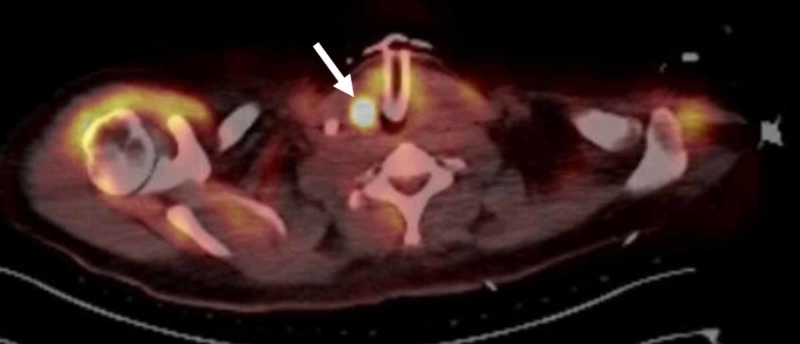
FDG-PET scan showing intensely hypermetabolic right thyroid nodule (white arrow) not detected on prior imaging scans to evaluate for occult malignancy. FDG-PET: Fluorodeoxyglucose positron emission tomography

Autoimmune workup was remarkable for elevated thyroid peroxidase (anti-microsomal) antibodies at 408 IU/mL (normal range <5.6 IU/mL) and thyroglobulin antibodies at 21.7 IU/mL (normal range <14.4 IU/mL). FDG-PET of the whole-body including brain demonstrated an intensely hypermetabolic thyroid nodule (Figure 2).

**Figure 3:**
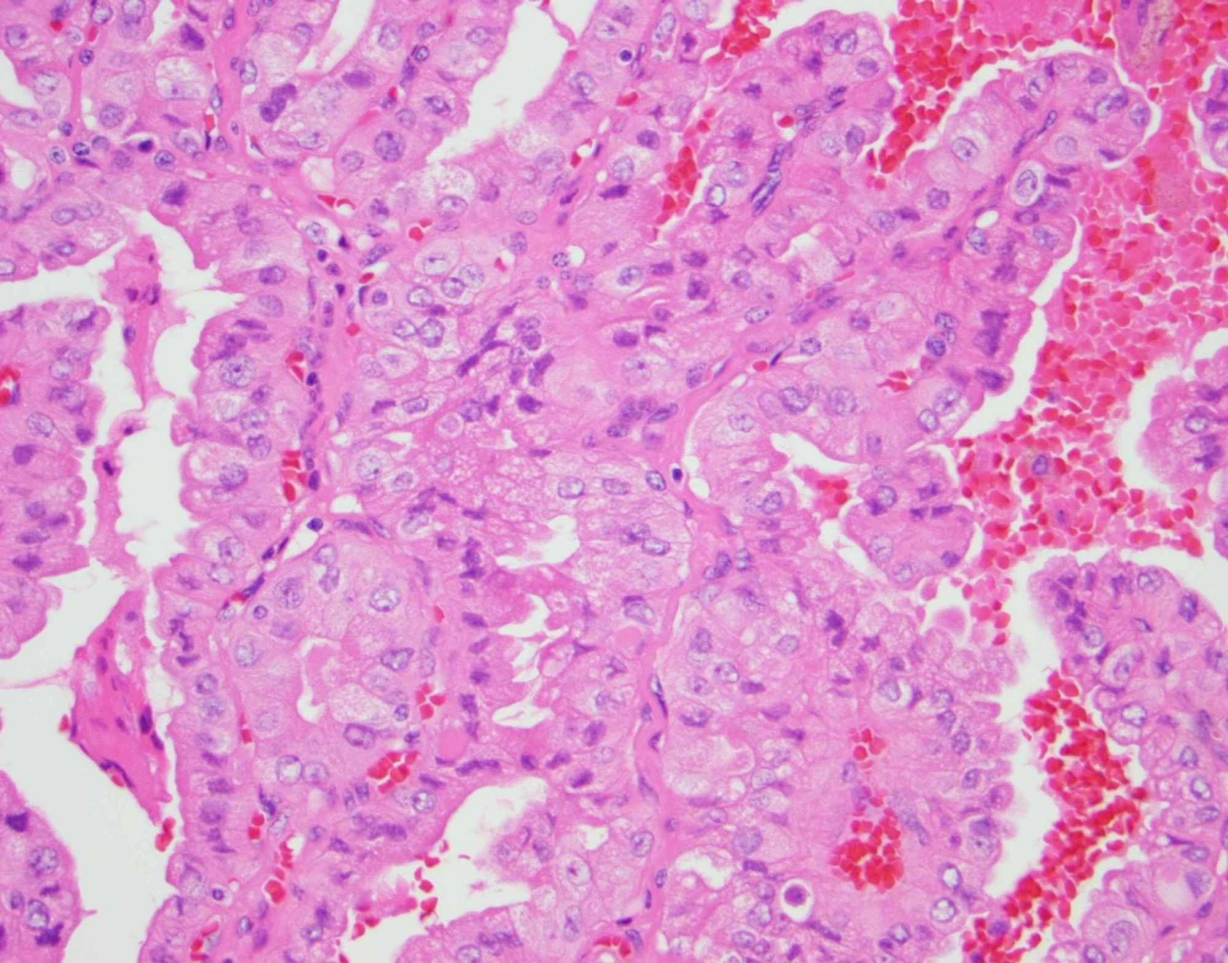
Pathology from fine needle aspiration. Right thyroid lobe (400x) showing polygonal cells, tall cells with basilar oriented nuclei, nuclear pseudoinclusions and eosinophilic cytoplasm consistent with tall cell papillary thyroid carcinoma.

She was started on 1000 mg IV methylprednisolone (IVMP). Serum NMDA titers were elevated at a dilution of 1:1280. She was also started on plasma exchange (PLEX). Despite IVMP and PLEX, there was no clinical improvement. Intravenous rituximab (700 mg) and intravenous immunoglobin (IVIG; 400 mg/kg/d x 5 days) were then administered with no improvement in clinical condition. She developed fluctuations in blood pressure, diabetes insipidus, and continued to have intermittent hyperthermia and near continuous dyskinesias. Fine needle aspiration of the hypermetabolic thyroid nodule was performed. She underwent total thyroidectomy which showed two foci of the tall-cell variant papillary thyroid carcinoma. Figure 3 shows tall cells with basilar oriented nuclei, nuclear pseudoinclusions, and eosinophilic cytoplasm consistent with tall cell papillary carcinoma. NMDA staining of the tissue was nonreactive.

Over the next following weeks, there was a gradual decrease in her dyskinetic movements and seizure activity. She was weaned from ventilator support to tracheostomy collar. Her hyperthermia and autonomic instability improved. Repeat serum NMDA titers were <1:12. She was discharged to a long-term acute care hospital (LTACH). Her discharge antiepileptic regimen included gabapentin 600 mg q8 hours, levetiracetam 1500 mg every 8 hours, phenobarbital 130 mg every 8 hours, and clonazepam 1 mg every 8 hours.

At follow-up visit approximately eight months later, her tracheostomy had been removed. She is ambulating, speaking with mild dysarthria, oriented to self, place, date, eating, and swallowing with the plan to transition to home from the nursing home. Her antiepileptic regimen at this time included lacosamide 200 mg q12 hours, levetiracetam 1500 mg q12 hours, phenobarbital 65 mg every 12 hours, and phenytoin 100 mg q12 hours.

## Discussion

We report a case of NMDA encephalitis concomitantly occurring in a patient with newly found papillary thyroid carcinoma. NMDA encephalitis typically affects younger individuals with a female preponderance [1]. In about 36% of cases an associated malignancy is found. This number approaches 58% in women between 18 and 45 years of age [1,8]. None report thyroid-associated tumor [9]. Our case confirms the lack of association between thyroid carcinoma and NMDA receptor encephalitis; however, it highlights the importance of neoplastic workup in patients with NMDA receptor encephalitis. The most common NMDA encephalitis-related tumor is an ovarian teratoma [1]. Others include lung cancer (small cell and adenocarcinoma), breast cancer, uterine adenocarcinoma, mature cystic teratoma of the fallopian tube, testicular tumor, thymic carcinoma, metastatic melanoma, gastric carcinoma, pancreatic cancer, colorectal cancer, prostate cancer, sex-cord stromal, neuroendocrine tumor, hepatic neuroendocrine carcinoma, pancreatic neuroendocrine, and Hodgkin lymphoma [8].

Recommendations for the treatment of NMDA encephalitis suggest first-line immunosuppression using any combination of intravenous steroids, intravenous immunoglobulin, and/or plasmapheresis [1,10]. Screening also is necessary given the association with tumors [5,6]. If a tumor is identified, removal is recommended. If a tumor is not found, screening has been recommended every six months for approximately four years [1,5-6]. Second-line therapy for NMDA encephalitis includes B and T cell-mediated immunotherapy with rituximab and/or cyclophosphamide [10]. Earlier treatment has been associated with better outcome and a majority of patients have good response to first-line therapy. Recent literature has suggested a role for bortezomib to target plasma cells in NMDA encephalitis refractory to the initial therapies [11].

In our patient, MRI brain and standard malignancy screening were negative. Only FDG-PET revealed evidence of a cancerous thyroid nodule. Even after second-line therapy with rituximab, she continued to worsen clinically (hyperthermia, diabetes insipidus, dyskinesias, and seizures). Her clinical and serological studies were improved after tumor removal likely highlighting the coincidental occurrence.

Importantly, papillary thyroid carcinomas are typically indolent [12]. However, the tall cell variant is aggressive causing vascular invasion, extrathyroidal extension, lymph node metastasis, and distant metastasis. It is characterized by height greater than twice its width, eosinophilic cytoplasm, basilar oriented nuclei [12]. Neurological involvement from this cancer variant has not been established. This case highlights the coincidental diagnosis of tall cell papillary thyroid carcinoma in a patient with newly diagnosed NMDA receptor encephalitis.

## Conclusions

We describe the coincidentally co-occurrence of NMDA encephalitis in a patient with papillary thyroid carcinoma. This case highlights the importance of presumed cases of non-paraneoplastic NMDA encephalitis, FDG-PET may help in detecting occult malignancies.
